# Surface-enhanced Raman scattering of water in aqueous dispersions of silver nanoparticles

**DOI:** 10.3762/bjnano.12.40

**Published:** 2021-05-25

**Authors:** Paulina Filipczak, Krzysztof Hałagan, Jacek Ulański, Marcin Kozanecki

**Affiliations:** 1 Department of Molecular Physics, Faculty of Chemistry, Lodz University of Technology, Zeromskiego 116, 90-924 Lodz, Poland

**Keywords:** Dynamic lattice liquid (DLL) simulations, liquid water, plasmons, resonance Raman effect, surface-enhanced Raman scattering, water structure

## Abstract

The resonance Raman effect (RRE) is a phenomenon which results in a strong selective enhancement of Raman signals from the samples. Previous studies showed that the RRE in liquid water directly corresponds to its supramolecular structure. It was also reported that the electric-field-induced orientation of water molecules on the electrode surface results in the surface-enhanced Raman scattering (SERS) effect. In this work, we show the SERS effect for water molecules in the dispersion of silver nanoparticles (AgNPs) without any external electrical field. An enhancement factor was estimated to be (4.8 ± 0.8) × 10^6^ for an excitation wavelength of 514.5 nm and for AgNPs with an average size of 34 ± 14 nm. The temperature experiment results showed a higher enhancement with temperature increase. Performed simulation studies revealed a slowdown of the mobility of the water molecules close to the surface of AgNPs.

## Introduction

What is the structure of water? This question is among the 125 most important unanswered questions of mankind and it was proposed by the prestigious Science Magazine [1]. Water is the most common compound in the world and a vital substance for every living organism. Despite being so abundant, water is still not an entirely known substance [2–3]. Raman spectroscopy is a very useful technique to study the water structure and molecular interactions in liquid water [4]. Analyses of Raman spectra of water in the OH stretching vibration region can give an idea of the dynamic supramolecular structure of water. There are many models of water structure in the liquid phase. These are generally grouped into two types: models with a continuum of geometric and energetic states (assuming tetrahedral coordination of water molecules) and models of water as a mixture of discrete species (water clusters) [5–8]. It is difficult to choose between them because the spatial network of hydrogen bonds has features of both continuous and discrete models. The broad Raman band of water located in the range of 3000–3800 cm^−1^ is a combination of a few overlapping components related to symmetric and antisymmetric H–O–H stretching, bending overtone, and free OH stretching vibrations [9]. Thus, a straight interpretation of the Raman spectrum of liquid water and its deconvolution is still a subject of discussion [9–12]. The problem of the liquid water spectrum deconvolution is crucial, because the ratio of integral intensities of bands with maxima at approx. 3200 and 3400 cm^−1^ is regarded as a measure of the water supramolecular structure [13–14]. The component with a maximum at approx. 3200 cm^−1^ is usually assigned to strongly hydrogen-bonded water molecules, or the so-called locally structured water, while the band at 3400 cm^−1^ is attributed to loosely bonded water molecules. Most of the authors distinguish three [15–16] or four [13] components of the band in the OH stretching vibration region. Irrespective of how the spectra are deconvoluted, all models of liquid water agree that the lower the frequency of the band, the stronger the hydrogen bonds and water structuration.

The interactions with various solutes [17–18] and the influence of the temperature [13] on the water structure can be analysed by the changes in the Raman spectra of water. Two types of agents can be distinguished: destructive agents (e.g., most ions, temperature, polar molecules) and structure-ordering agents (e.g., apolar molecules, biological macromolecules, or hydrophobic surfaces [19]). The intensity of the OH stretching vibration band related to structured water depends also on the excitation wavelength, due to the resonance Raman effect. The first report of this phenomenon was presented by Pastorczak et al. [20]. It was shown that the 3200 cm^−1^ band is in resonance with the light in the red range of the spectrum, due to the presence of vibrational overtones and combinational modes in the UV–vis absorption spectrum of liquid water. Woutersen and Bakker [21] showed that fast intermolecular transfer of vibrational energy is present only in structured systems, such as the hydrogen bond network in liquid water, which favours the presence of resonance. This indicates that OH oscillators of water molecules can reach the resonant state provided that they are strongly linked to each other via hydrogen bonds. All factors considered destructive to the hydrogen bond structure (e.g., temperature) make the resonance effect weaker [20].

Silver nanoparticles (AgNPs) are gaining more and more popularity in various applications, such as electronics [22], photonics [23], and medicine [24]. Silver nanocolloids are also commonly used as an enhancing substrate in surface-enhanced Raman scattering (SERS) [25–26]. Since the discovery of the SERS effect in 1974 [27], most of the research is focused on biomolecules and medical applications [28–29]. The SERS effect of water has been studied since 1980. There are reports on the SERS effect of water in aqueous solutions of electrolytes placed between metal electrodes (Ag, Au, Cu) under an applied negative potential [30–32]. In these studies, special surface complexes involving “metal adatoms (clusters), halide ions, cations, and water” were responsible for the observed SERS effect [32–33]. The spectra presented there were different from the typical Raman spectrum of liquid water, with only a weak band at approx. 3400 cm^−1^ present in the OH stretching vibration region. Additional factors, such as confinement effects or the presence of some solutes, are common in the research of the SERS effect of water [33–34]. In our previous work, we compared, for the first time, spontaneous and stimulated Raman surface enhancement of the signal of liquid water in an aqueous dispersion of silver nanoparticles [35]. High enhancement factors (in the magnitude of 10^6^) were obtained for the results from both techniques. In this work, further investigations on the SERS effect for water molecules in aqueous dispersions of AgNPs are presented. The influence of temperature on the enhancement effect is discussed with respect to the supramolecular structure of water.

## Results and Discussion

### Characterisation of silver nanoparticles

**Figure 1:**
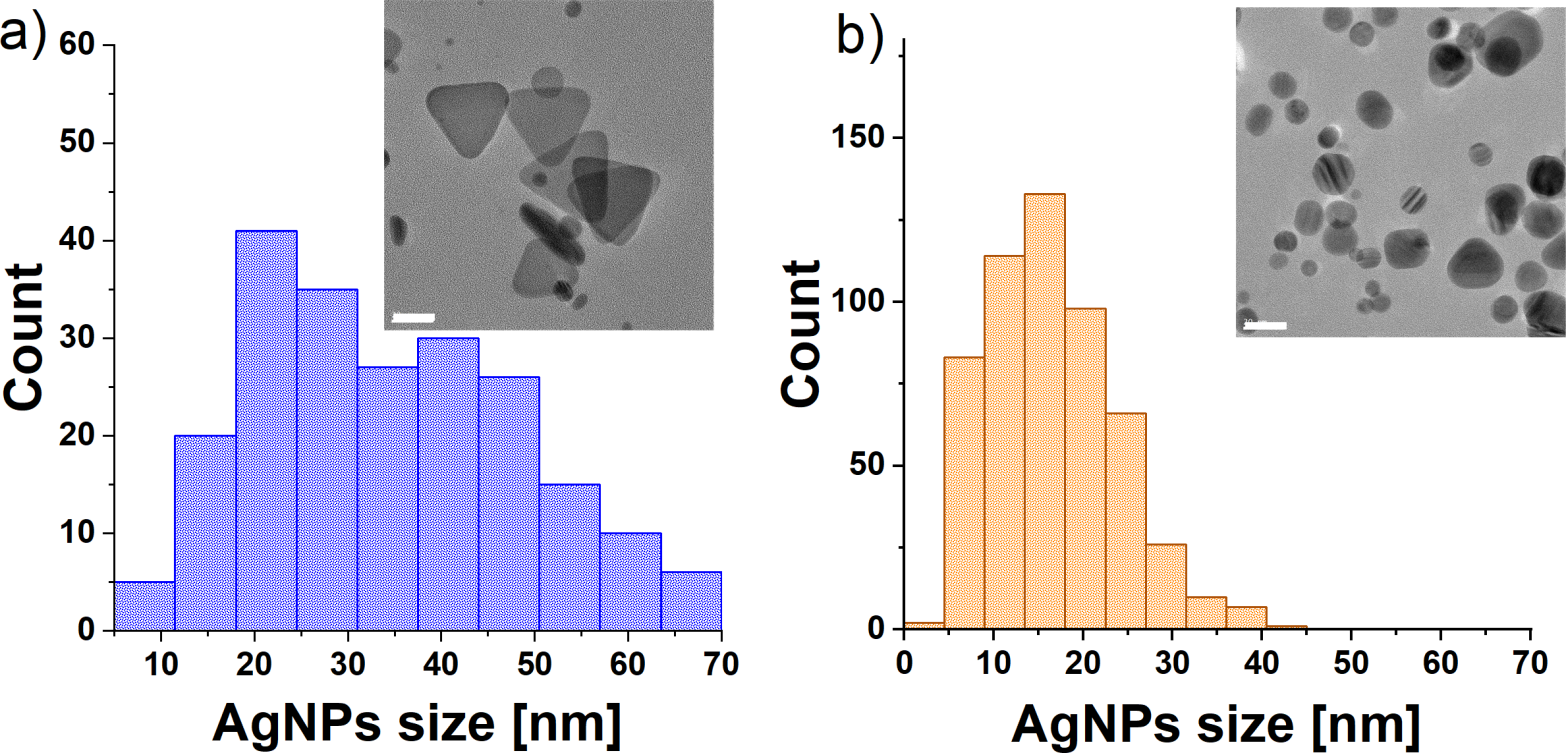
Size distributions and representative TEM images of AgNPs samples (white scale bar is 20 nm): (a) AgNPs blue and (b) AgNPs yellow. Experimental data from [35].

This article presents the SERS effect of water in aqueous dispersions of AgNPs. The AgNPs were synthesized according to the protocol proposed by Frank et al. [36]. Two types of nanoparticles were obtained: AgNPs blue and AgNPs yellow, which were labelled according to the colour (compare the UV–vis spectra shown in Figure S1 in Supporting Information File 1). High-resolution transmission electron microscopy (TEM) analysis was performed to estimate the size and identify the shape of the nanoparticles (see Figure 1).

The nanoparticles in the AgNPs blue sample had a triangular prism shape with an estimated average size of 34 ± 14 nm, while in the AgNPs yellow sample, the shape of the AgNPs was quasi-spherical with an average size of 16 ± 7 nm. The sizes of the nanoparticles were estimated based on the diameter of circumscribed circles on the AgNPs – see Section SI3 in the Supporting Information section of [35].

### Surface-enhanced Raman scattering effect of water

**Figure 2:**
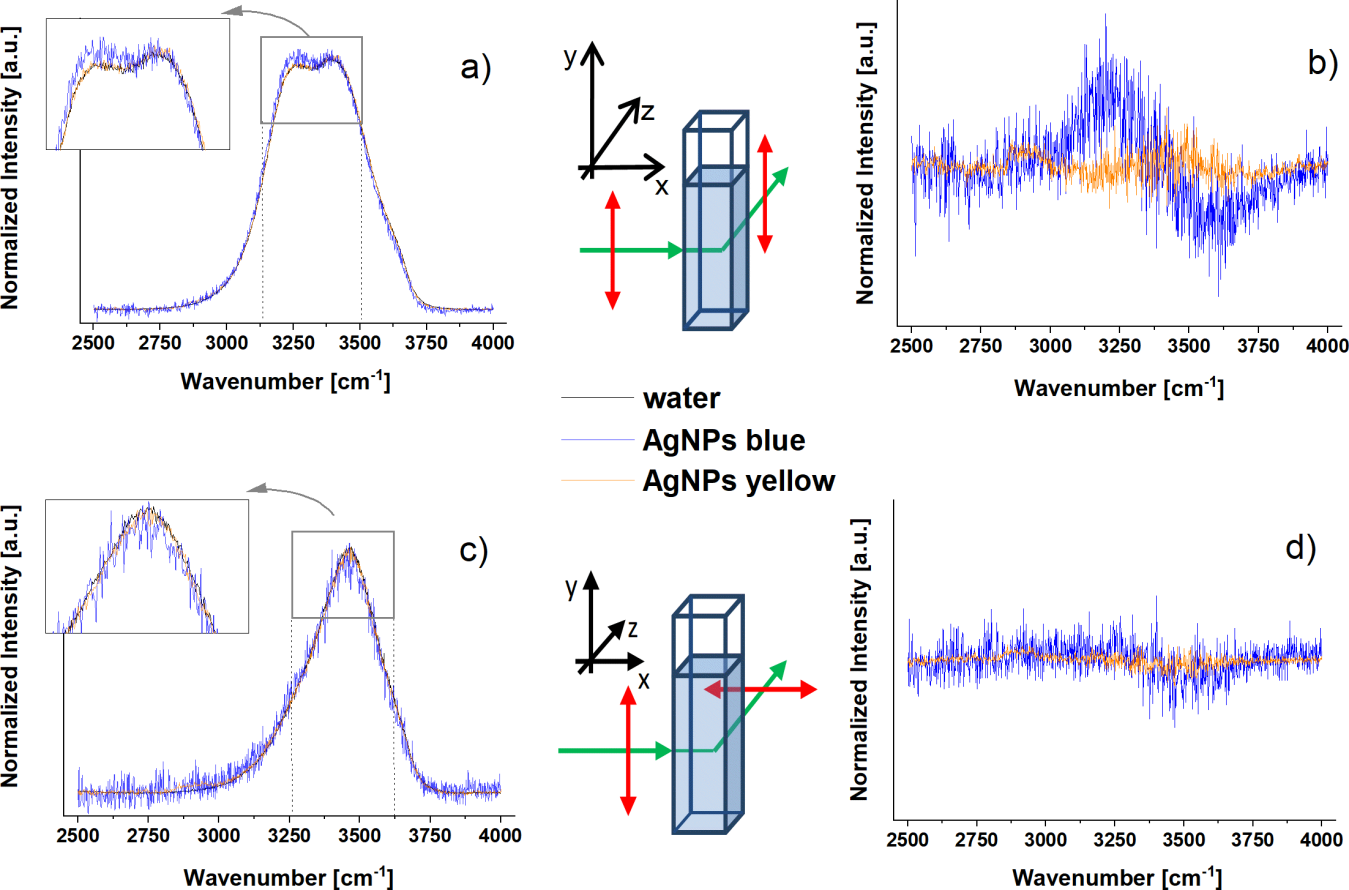
Polarised Raman spectra in the OH stretching vibration region for pure water and two AgNPs dispersions acquired with parallel (a) and perpendicular (c) configurations, respectively. Panels (b) and (d) show differential spectra of AgNPs relative to the water spectrum. The inserts show a spectral region zoomed in on the maxima of the OH stretching bands.

Polarised Raman spectra for pure water and aqueous dispersions of silver nanoparticles were performed at room temperature (see Figure 2). Polarised Raman measurements were carried out in x(y,y)z (parallel) and x(y,x)z (perpendicular) configurations. All spectra were normalised to the total integral intensity. Additionally, the differential spectra obtained by subtracting the water spectrum from the spectra of AgNPs dispersions are shown.

The enhancement of the low wavenumber component in the spectrum acquired in the parallel polarization configuration (polarized OH stretching mode), located at 3200 cm^−1^, is visible in comparison to pure water only in the blue sample case. This is an unexpected Raman effect as the presence of additives in liquid water usually results in a decrease of the intensity of the 3200 cm^−1^ line, which is explained by a reduction in the number of water–water hydrogen bonds in the system and a decrease in the strongly structured water fraction. The observed increase in the intensity of the 3200 cm^−1^ band in the spectrum of the AgNPs blue sample, in comparison to the pure water spectrum, may be regarded as a subtle effect. However, this result is reproducible as three independently synthesized AgNP dispersions were tested. Moreover, it is necessary to underscore that the concentration of AgNPs is very low – 1.5 nmol/dm^3^ (this value corresponds to the molar concentration of nanoparticles, not to the concentration of silver).

This phenomenon cannot be a result of the presence of ionic species in the dispersion (e.g., citrate, sodium, potassium, bromide – only in the AgNPs yellow sample) because ionic additives result in the decrease of the low frequency component around 3200 cm^−1^ due to their destructive impact on the hydrogen bond network in liquid water [17]. Moreover, the observed phenomenon is present only in the AgNPs blue sample, not in the AgNPs yellow sample. Also, changes in the structure of AgNPs induced by laser irradiation may be excluded, as an identical UV–vis absorption spectra of the investigated AgNPs dispersions (see Figure S1 in Supporting Information File 1) collected before and after Raman measurements confirmed their stability under the measurement conditions (the photo- and thermal stability of the investigated samples was discussed in [37]).

**Figure 3:**
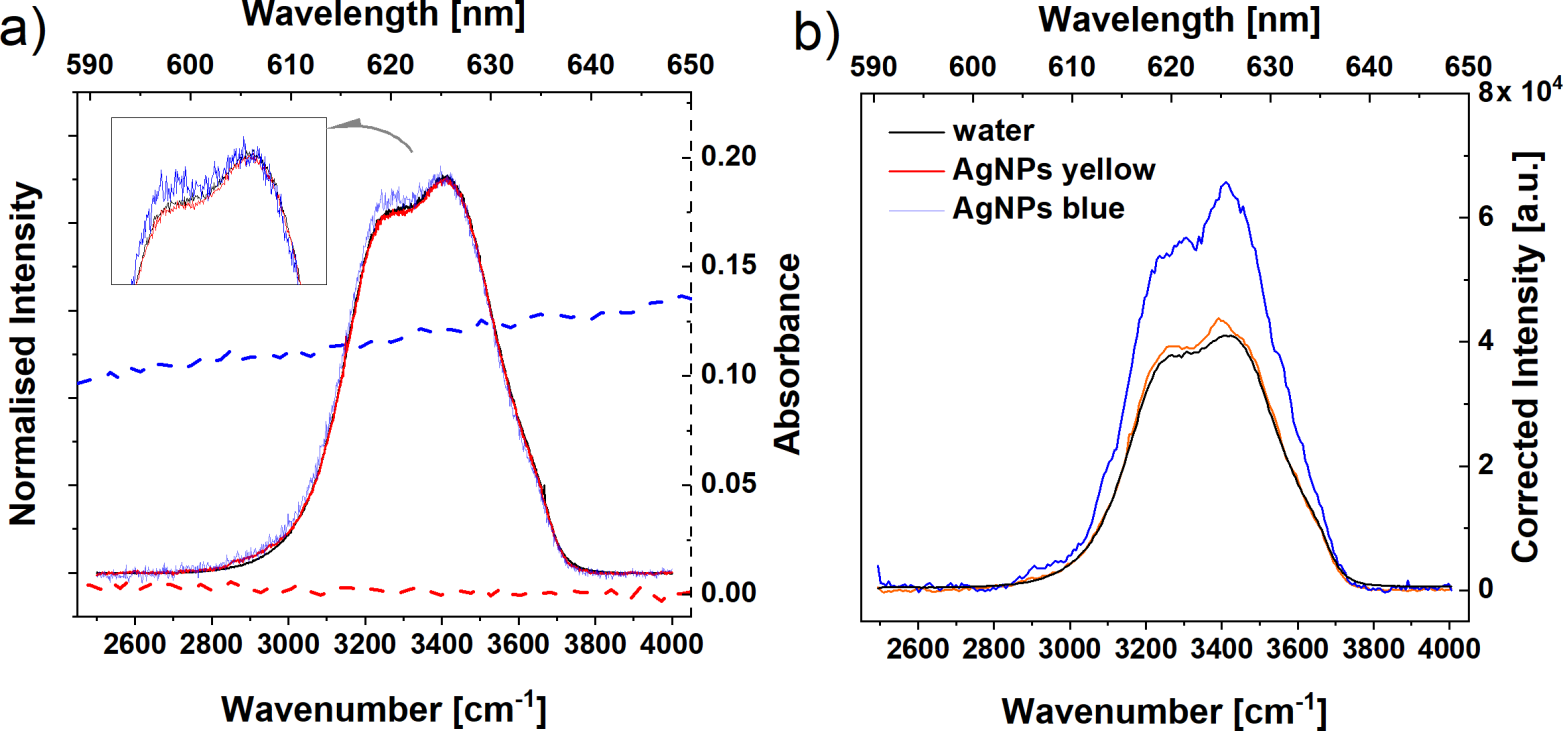
(a) Normalised Raman spectra in the OH stretching vibration region for pure water and two AgNPs dispersions, dotted lines are absorption spectra of AgNPs samples, inset shows a spectral region zoomed in on the maxima of the OH stretching vibration band. (b) The same Raman spectra after reabsorption correction – figure adapted from [35].

The observed enhancement effect could be explained by (i) differences in the selective light absorption in the visible part of the spectrum among studied samples, (ii) by the surface enhancement effects for water molecules in the neighbourhood of metal nanoparticles, or (iii) by the “structure-ordering” character of AgNPs for water. Some of the reports show that the water structure forms a specific conformation on the metal surfaces, which is very different in comparison to bulk water [24]. It was explained that the ordering of water molecules near to AgNPs is due to the electrostatic interactions between water and AgNPs. Some simulation studies also reported the enhancement of hydrogen bonds in the water structure near gold nanoparticles protected by various ligands [38–39]. The results of hydrogen bond dynamics and calculated far-IR spectra showed that a well-defined multilayered structure of water is formed close to the surface of the metal nanoparticle. The stabilization of this structure may be additionally enhanced by the mobility decrease in the nearest vicinity of the metal nanoparticle and by the increase of the rotational relaxation time and residence time of water molecules surrounding the ion wall in a charged monolayer-protected Au nanoparticle [39]. Assuming that the observed Raman enhancement is only a result of the specific orientation of water molecules on a silver surface, one should expect that this effect is visible for all AgNPs and is stronger for samples with a higher total metal surface. Taking into account the smaller average size of the AgNPs in the AgNPs yellow sample (higher total surface of metal NPs) in comparison to the AgNPs blue sample, a stronger Raman enhancement should be measured in the AgNPs yellow sample. As it is not true, the hypothesis that the orientational effects are the only ones responsible for the observed effect may be rejected. Of course, it does not mean that the water molecules could not be specifically organised onto the metal surface. Therefore, the shape and size of AgNPs may be the crucial point – more precisely, the optical plasmons responsible for the colour of the AgNPs. The location of the enhancement of the electric field around the nanoparticle is also an important issue. It is shown that a strong electric field enhancement takes place on the edges and on the tips of a triangular nanoscale prism (in this case, the AgNPs blue sample) in comparison to a low enhancement on a spherical surface (AgNPs yellow sample) [40]. To verify the influence of selective light absorption by different samples on the Raman spectrum, the reabsorption correction procedure was performed (details of the data treatment are described in the Section SI1 of the Supporting Information of [35]). The Raman signal of the AgNPs samples was corrected by the transmittance values of AgNPs in the OH stretching vibration spectral region. Also, the transmittance value at the excitation wavelength used in the Raman experiments (514.5 nm) was taken into account. After this correction, the spectra were then treated with a baseline correction. Only the non-polarised Raman spectra were analysed further to eliminate additional influence of the polarisers on the final (measured) signal. As AgNPs dispersions have non-zero absorption in the spectral ranges of both the Raman pump (514.5 nm) and Raman scattered light (in the range of 610–635 nm; for the absorption spectra of the samples, see Figure S1 in Supporting Information File 1), the Raman spectra should be corrected due to the reabsorption process (see Section SI1 in the Supporting Information section of [35]). Normalised Raman spectra and the same data after correction for AgNPs spectra are presented in Figure 3.

As Figure 3b presents, the spectrum of the AgNPs blue sample after correction shows a significantly higher increase in intensity in comparison to those of water and AgNPs yellow (only a very small increase in the Raman intensity is observed). Moreover, the band related to the OH stretching modes of water changes its shape. The intensity of the component at 3400 cm^−1^ becomes stronger than that at 3200 cm^−1^. In this form the enhanced Raman signal is very similar to other results reported for the resonance water spectrum [34]. Moreover, in the case of the AgNPs blue sample, the excitation laser light is absorbed much more strongly than in pure water or in the AgNPs yellow sample. The absorption of laser light in the AgNPs blue sample leads to the excitation of both plasmons in the AgNPs and vibrations of water molecules. Thus, one can conclude that the most probable hypothesis is that the enhancement of the Raman signal due to the OH stretching modes is related to the SERS effect of water molecules in the presence of metal nanoscale objects. The detailed discussion about the lack of enhancement in the spectrum of AgNPs yellow sample is described in our previous work [35].

The maximal number of water molecules interacting directly with the surface of AgNPs was estimated based on the size and shape of AgNPs in the AgNPs blue sample, which was determined by TEM (Figure 1), and on the Ag concentration in the dispersion (17.96 µg/mL), which was determined by flame atomic absorption spectrometry (FAAS). It was found that only one water molecule per 10 million is in direct contact with the surface of AgNPs. It is also worthy to mention that the model used to calculate the part of the water molecules in the direct vicinity of AgNPs yielded an overestimated value (the detailed calculations are described in the Section SI3 in the Supporting Information section of [35].).

The classic enhancement factor (EF) for a given substrate is given by the formula [41]:

[1] $$\text{EF}=\frac{I_{\text{SERS}}/N_{\text{surf}}}{I_{\text{RS}}/N_{\text{vol}}},$$

where *I*_SERS_ and *I*_RS_ denote the intensity of the SERS signal and the normal Raman scattering signal, respectively. The values *N*_surf_ and *N*_vol_ are the average molecule numbers in a scattering volume for the SERS experiment conditions and for Raman scattering, respectively. The studied system herein is not a typical SERS system regarding the SERS substrate and the analysed substance (target molecule) in a low concentration. In our system, the solvent is simultaneously an analyte and it is in a much higher concentration than the substrate itself. However, a stronger SERS effect could be induced by the water molecules in the nearest vicinity of the Ag surface. Therefore, the number of water molecules directly interacting with AgNPs was estimated and used to calculate the EF. As all measurements were performed under the same conditions, all calculations, for simplicity, were done for 1 dm^3^ of the samples (water and AgNPs blue dispersion). The enhancement factor for AgNPs blue was calculated by using Equation 1 as follows: *I*_SERS_ and *I_RS_* are integral intensities for AgNPs blue and water spectra, respectively (AgNPs blue spectrum after the reabsorption correction) and *N*_surf_ and *N*_vol_ are the numbers of water molecules located on the surface of AgNPs and water molecules in pure water in 1 dm^3^, respectively (for the calculations of these values, see section SI3 in the Supporting Information section of [35]). The calculated EF is equal to (4.8 ± 0.8) × 10^6^, taking into account the size distribution of the nanoparticles in the AgNPs blue sample. This result assumes that all water molecules located on the Ag surface participate in the resonance effect. Of course, such a strong EF may be registered only if the electromagnetic mechanism of enhancement occurs. By including the subsequent layers of hydration in the calculations, as water molecules participate in the SERS effect (*N*_surf_), we obtain a lower EF value; however, still in the order of 10^6^.

### Influence of temperature

**Figure 4:**
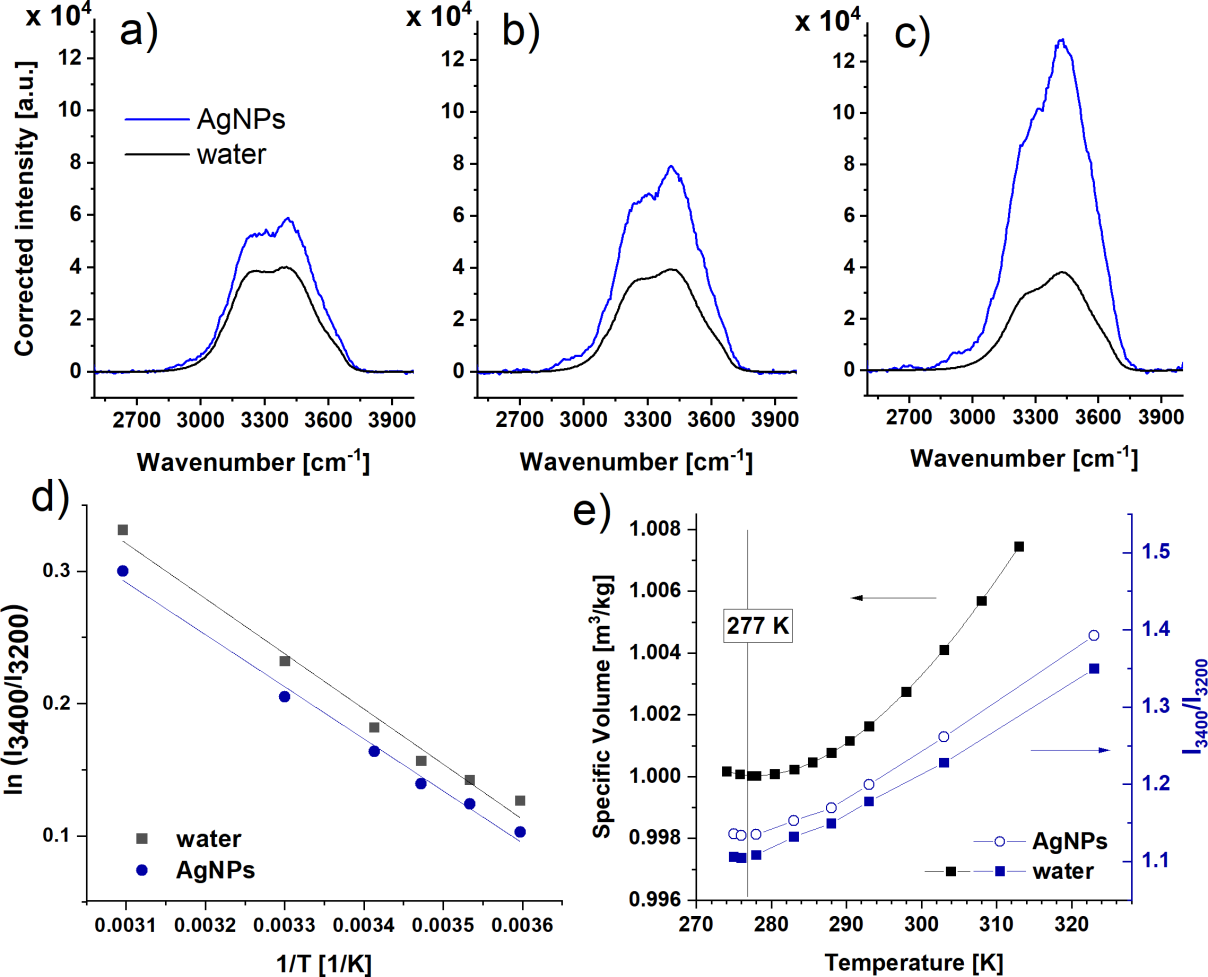
Non-polarized Raman spectrum of water and reabsorption corrected spectrum of AgNPs blue sample at (a) 276 K, (b) 293 K, and (c) 323 K. (d) Arrhenius plot of the ratio Ri = *I*_3400_/*I*_3200_ for water and AgNPs blue. (e) Dependence of a specific volume with temperature for water and a comparison of ratios Ri = *I*_3400_/*I*_3200_ for water and the AgNPs blue sample.

The SERS effect is strictly related to the interactions of the analyte and the metal plasmons and thus it is temperature sensitive [42–45]. Thus, the Raman measurements were carried out at different temperature values to check how the temperature influences the observed SERS effect. A comparison between Raman spectra of water and AgNPs blue after the reabsorption correction, measured at selected temperature values, is presented in Figure 4a–c.

Figure 4a–c show, as expected, a decrease of the low-frequency component (band at approx. 3200 cm^−1^) in relation to the high-frequency component (band at approx. 3400 cm^−1^) with a temperature increase for both water and AgNPs blue sample. Although the trends of the spectra of pure water and the AgNPs blue sample are the same, the resonance effect changes with temperature – it significantly increases with a temperature increase. The increase of the SERS intensity with a temperature increase was observed also in the SERS studies of painting materials on AgNPs substrates [43]. In our system, it can be suggested that, at a low temperature, water–water interactions dominate resulting in a weak SERS effect. Conversely, at a higher temperature, these interactions weaken and the water molecules favourably interact with the metal surface causing an increase of the SERS intensity in the OH stretching vibration region. To quantitatively compare the temperature effect on the observed phenomenon, the ratio of the intensities (signal amplitudes) of high (*I*_3400_) and low (*I*_3200_) frequency components were compared. Figure 4d presents the Arrhenius plot of Ri = *I*_3400_/*I*_3200_ for water and AgNPs sample spectra after reabsorption correction. The data were treated by linear fitting (solid lines), according to the formula:

[2] $$\ln\left(\frac{I_{3400}}{I_{3200}}\right)=\ln\left(A\right)-\frac{E_{\text{a}}}{RT},$$

where *A* is a pre-exponential factor and *R* is the universal gas constant. This data treatment is analogous to the procedures proposed by Walrafen et al. [46] for the calculation of the enthalpy Δ*H*° referring to the formation of O–H∙∙∙O units. The results presented here are consistent with the results proposed by Walrafen et al. (see Figure S2 in Supporting Information File 1), which confirms a correct approach to the data analysis. The slopes for the dependencies presented in Figure 4e are −394 ± 18 and −417 ± 25 K for water and the AgNPs sample, respectively. Calculated activation energies *E*_a_ are equal to 3.3 ± 0.1 kJ/mol for water and 3.5 ± 0.2 kJ/mol for the AgNPs sample (*E*_a_ calculated for water from the results presented by Walrafen is equal to 3.1 ± 0.2 kJ/mol). This energy is related to conformational changes in the water structure due to the redistribution of hydrogen bonds due to the temperature increase. In other words, *E*_a_ is the energy of transition between a state where a strong hydrogen bond structure dominates to a state where the number of hydrogen bonds is much smaller. A larger value of *E*_a_ for the AgNPs dispersion in comparison to pure water is an effect of water structuration due to the presence of AgNPs. Therefore, one can simply conclude that more energy is needed to destroy the water structure in the AgNPs dispersion than in pure water.

### Simulation studies

Dynamic lattice liquid (DLL) simulation studies based on the face-centred cubic (FCC) lattice approach were performed, in which averaged values (average of total energy values of the interactions with all neighbouring elements) were taken into account without a detailed differentiation of the particular elements [47–50]. Each lattice site represented a solvent, here a water molecule or a Ag atom. Two states of water were distinguished: (i) bulk water, in which each water molecule is surrounded only by other water molecules and (ii) water close to the Ag surface (an immobile wall representing a fragment of AgNP), in which a water molecule can form a maximum of three H bonds with other water molecules and one of its electron-donor or electron-acceptor centres is involved in the interaction with the Ag surface [51]. To bring the Monte-Carlo simulations closer to a real system, short-range interactions between the nearest neighbours were also assumed besides the movement rules defined by the DLL algorithm itself (providing an excluded volume interaction). They were characterized by two types of interaction energy: between water and water (ε_ww_), and between Ag and water (ε_aw_). It means that the total ε being a potential energy barrier for the movement of a given element is defined by the surroundings of this element [52]. The probability of a successful solvent displacement (reflecting the water diffusion) was defined by the Boltzmann distribution:

[3] $$P=e^{\frac{-E}{RT}},$$

where *T* is the temperature (set as 300 K), *R* is the universal gas constant, and *E* could take two values: *E*_w_ (=Σ^3^_k=1_ ε_ww +_ ε_aw_) close to the wall or *E*_b_ (=Σ^4^_k=1_ ε_ww_) far from the wall, where all the neighbours of a given element were water-type. A summation to four reflects a possible number of hydrogen bonds for one water molecule. The values *E*_w_ and *E*_b_ were estimated taking into account different approaches and in all cases the interaction energy values were rescaled to the given level of *E*_b_.

**Figure 5:**
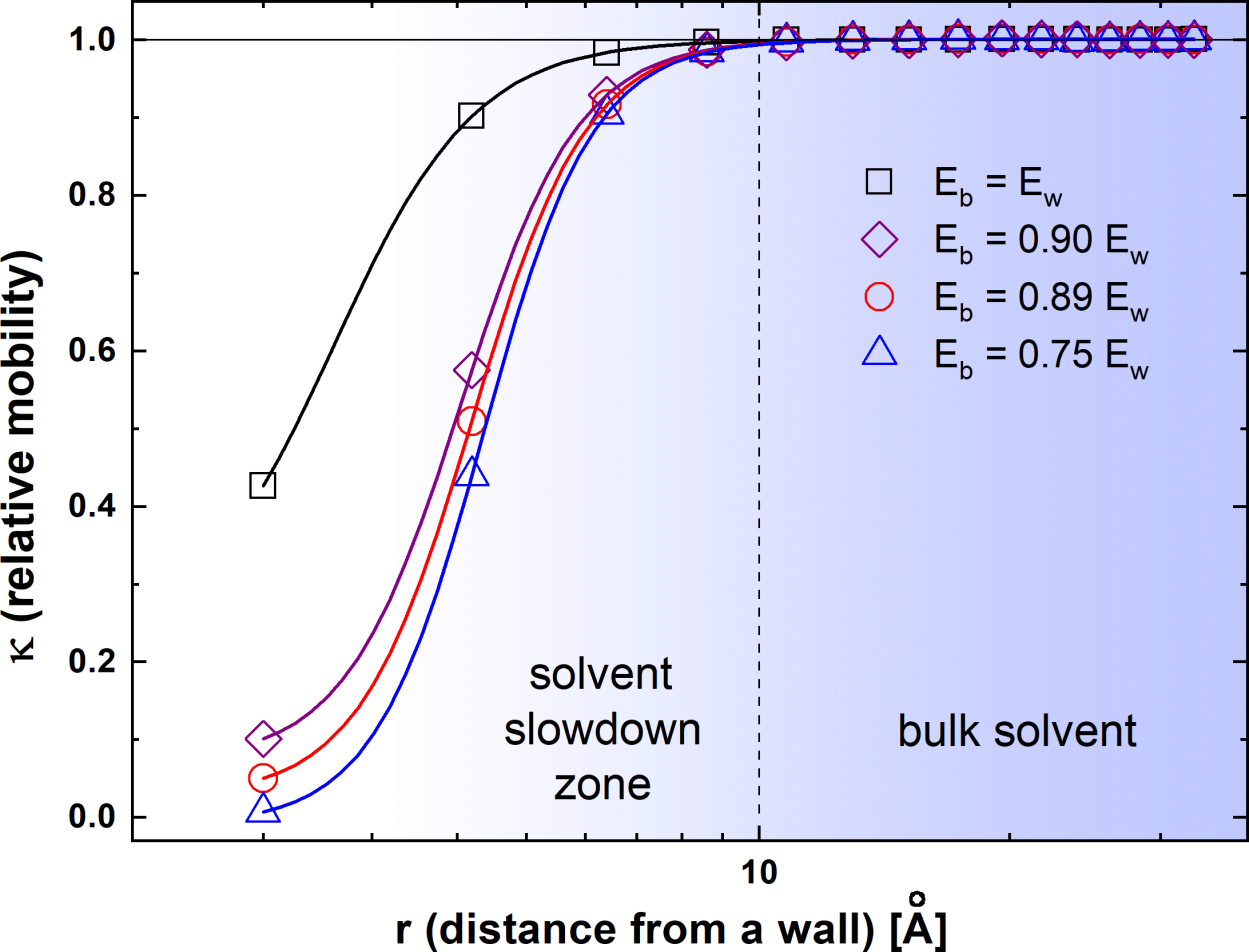
DLL simulation results. The relative slowdown of the solvent mobility expressed by the κ parameter (see Equation 4) as a function of the distance from the Ag surface for different variants of intermolecular interactions: black squares −*E*_b_ = *E*_w_; red circles −*E*_b_ = 26 kJ/mol, *E*_w_ = 29.3 kJ/mol (*E*_b_ = 0.89*E*_w_); blue triangles −*E*_b_ = 22.9 kJ/mol, *E*_w_ = 30.4 kJ/mol (*E*_b_ = 0.75*E*_w_); purple rhombuses −*E*_b_ = 19 kJ/mol, *E*_w_ = 21.1 kJ/mol (*E*_b_ = 0.90*E*_w_). The error bars are smaller than the symbols used.

The absolute values of ε_ww_ and ε_aw_ are practically inaccessible in an experiment. Thus, five other variants were considered (a detailed description of the assumptions and reasons for considering these variant can be found in Supporting Information File 1): (i) *E*_b_ = *E*_w_ (athermal case, in which all interactions are equal – CASE 0); (ii) *E*_b_ = 26 kJ/mol, *E*_w_ = 29.3 kJ/mol (*E*_b_ = 0.89*E*_w_) – CASE A; (iii) *E*_b_ = 22.9 kJ/mol, *E*_w_ = 30.4 kJ/mol (*E*_b_ = 0.75*E*_w_) – CASE B; (iv) *E*_b_ = 17.5 kJ/mol, *E*_w_ = 16.6 kJ/mol (*E*_b_ < *E*_w_ which corresponds to athermal conditions – *P* = 1 – and it was not considered further) – CASE C; (v) *E*_b_ = 19 kJ/mol, *E*_w_ = 21.1 kJ/mol (*E*_b_ = 0.90*E*_w_) – CASE D. The solvent mobility was calculated as an averaged probability of movement of a water-like element at a given distance (*r*) from the Ag wall. The Figure 5 presents the results of the DLL simulation. The κ parameter is defined as:

[4] $$\kappa \left(r\right)=\frac{\text{water mobility at a distance }r\text{ from the Ag wall}}{\text{water mobility in the bulk}}$$

and it represents the relative slowdown of the solvent mobility induced by the presence of a fixed wall in relation to the solvent mobility in the bulk (determined for a solvent in a non-confined space).

The simulation results show that even under athermal conditions (*E*_b_ = *E*_w_ – case without electrostatic interactions), the solvent mobility is lower near the surface in comparison to the bulk. The stronger the water interaction with the surface (in relation to water–water interactions in the bulk), the lower the solvent mobility close to the Ag surface. Moreover, this effect is not limited only to the first hydration layer, but it is also transferred to deeper layers of water up to approx.10 Å (this value is only slightly impacted by the interaction energies). As a result, water molecules around AgNPs have a lower kinetic energy and a longer dwell time that favours structure stabilization. A similar effect has been previously observed for polymer chains [49–50], even when only excluded volume interactions were assumed.

Previous works [38–39], as well as the performed simulations, support the finding presented herein in which silver nanoparticles are able to induce surface-enhanced Raman scattering of water molecules. The SERS effect is present only for water molecules that are in direct contact with AgNPs; however, the dynamic properties of water molecules close to the metal surface probably differ from the ones in the bulk up to a couple of hydration layers. This suggests that at least there might be a partial structuration of water in this region. Moreover, the presence of citrate ions adsorbed onto the surface of the AgNPs should favour the structuration of water molecules [53].

## Conclusion

The results presented here are a report on the SERS effect for water in aqueous dispersions of silver nanoparticles measured without an electric field. The increase in intensity of the lower wavenumber component in the OH stretching vibration mode, attributed to strongly structured water, has been observed for non-polarized as well as for parallelly polarized Raman spectra. These spectra were collected for aqueous dispersion of AgNPs, which exhibit a higher absorption in the region of Raman excitation wavelength (514.5 nm). The reported phenomenon is depended on the size and shape of AgNPs and on the wavelength of the surface plasmon, characteristic for particular AgNPs. The performed reabsorption correction on the spectra of the AgNPs blue sample showed a significant enhancement in the whole range of the OH stretching vibrational mode. Assuming that all the water molecules located on the Ag surface participate in the resonance effect, the calculated EF is equal to (4.8 ± 0.8) × 10^6^, taking into account the size distribution of nanoparticles in the AgNPs blue sample. The temperature analysis showed that the observed enhancement increases with the increase of the temperature. The calculated activation energy for a AgNPs sample is larger than that for pure water, which is an effect of water structuration around the AgNPs. Further molecular interpretation of the effects of this structure requires the adoption of a specific model of the water structure – continuum or cluster. The obtained results are consistent with the work from Pastorczak et al. [20], regarding the resonance Raman effect in liquid water, and data presented by Walrafen et al. [39]. They are also in agreement with the DLL simulation results presented herein as well as with the work from Cataliotti at al. [16] on the structure-ordering properties of AgNPs.

## Experimental

### Preparation of silver nanoparticles

Silver nanoparticles were synthesized via simple chemical reduction of silver nitrate with sodium borohydride [36]. The volume added of potassium bromide during the synthesis was crucial for the size control of AgNPs. The sample without added KBr turned blue and the ample with 40 µL of the added KBr turned yellow. The synthesized AgNPs were studied by UV–vis and Raman spectroscopy without any purification.

### Characterization of silver nanoparticles

The absorption spectra of AgNP dispersions were obtained by using double-beam UV–vis–NIR spectrophotometer (Cary 5000 - Varian) in the 200–800 nm range in relation to pure water, used as the reference sample.

The TEM images were obtained by using a Jeol ARM 200F high-resolution transmission electron microscope.

The silver concentration in the AgNP dispersion was determined by FAAS using the GBC 932 plus instrument. The calibration was made using the silver standard solution (received from Merck).

### Raman spectroscopy

The Raman spectra were obtained by using a T64000 (Jobin Yvon) triple-grating spectrometer (Ar laser excitation line – 514.5 nm) with a spectral resolution of approx. 0.5 cm^−1^. The measurements were carried out in a macro chamber, in a spectrofluorometric QX quartz cuvette in a classic illumination configuration – observation geometry (the excitation and scattered beams were perpendicular). The laser power on the sample was in range of 25–30 mW. The acquisition time was 2 × 30 s per spectral range, which gave 5 min for spectrum in the range of 2500–4000 cm^−1^. The temperature measurements were carried out by using a single cell Peltier accessory (Varian Inc.), which allows for temperature change of a liquid in the standard 10 mm pathway cuvette with an accuracy of ±0.1 °C.

### Simulation parameters

Experimental works were supported by Monte Carlo simulations performed by using the dynamic lattice liquid model. Detailed description of the DLL algorithm and its previous implementations for diffusion studies were presented elsewhere [47,49–50]. This model was chosen because it assumes movement cooperativity, which is crucial for water systems [4]. Moreover, simulations are faster than molecular dynamics methods.

The simulations were performed on a 100×100×32 FCC lattice and averaged over 10 000 time units. Two stiff immobile walls representing Ag {100} surfaces were placed at *z* = 1 and *z* = 32. In other directions, (*x*, *y*) periodic boundary conditions were used. All lattice sites were occupied either by water-like or by Ag-like elements. The size in the *z* direction was large enough to avoid finite-size effects [48]. A large size in *x* and *y* directions provided good spatial averaging for the results.

## Supporting Information

Supporting Information File 1 contains the absorption spectra of AgNPs samples (SI1), the Arrhenius plot for the data extracted from the work of Walrafen et al. (SI2), and the procedure used to estimate the energy barriers for the movement of water molecules in the bulk and close to the Ag surface (SI3).

File 1 Experimental data and simulation of the energy barriers for the movement of water molecules in the bulk and close to the Ag surface.
