# Supplementary material for: Surface-enhanced Raman scattering of water in aqueous dispersions of silver nanoparticles
Source: Beilstein J Nanotechnol. 2021 May 25;12:497–506. doi: 10.3762/bjnano.12.40 (PMC8182675; doi:10.3762/bjnano.12.40)
Supplement: File 1 — Experimental data and simulation of the energy barriers for the movement of water molecules in the bulk and close to the Ag surface. [file Beilstein_J_Nanotechnol-12-497-s001.pdf]

BEILSTEIN JOURNAL OF NANOTECHNOLOGY

Supporting Information

for

Surface-enhanced Raman scattering of water in aqueous dispersions of silver nanoparticles

Paulina Filipczak, Krzysztof Hałagan, Jacek Ulański and Marcin Kozanecki

Beilstein J. Nanotechnol. **2021,** *12,* 497–506. doi:10.3762/bjnano.12.40

Experimental data and simulation of the energy barriers for the movement of water molecules in the bulk and close to the Ag surface

License and Terms: This is a supporting information file under the terms of the Creative Commons Attribution License (https://creativecommons.org/licenses/by/4.0). Please note that the reuse, redistribution and reproduction in particular requires that the author(s) and source are credited and that individual graphics may be subject to special legal provisions.
The license is subject to the *Beilstein Journal of Nanotechnology* terms and conditions: (https://www.beilstein-journals.org/bjnano/terms)

SI1 Absorption spectra of AgNP samples

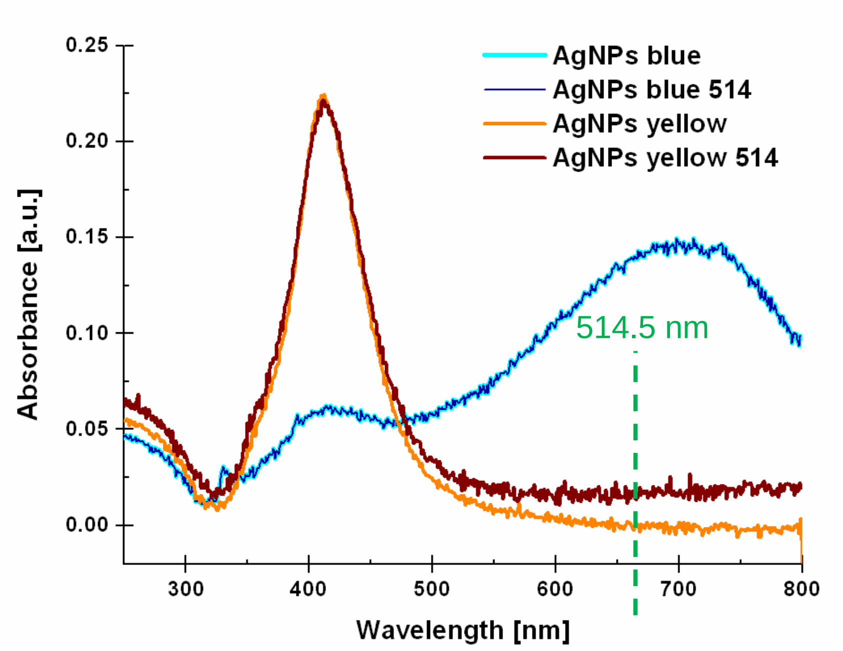

Figure S1: Absorption spectra of the studied water dispersions of AgNPs before and after (excitation wavelength - 514.5 nm was marked by dashed line) Raman measurement.

SI2 Arrhenius plot for data extracted from Walrafen et al.

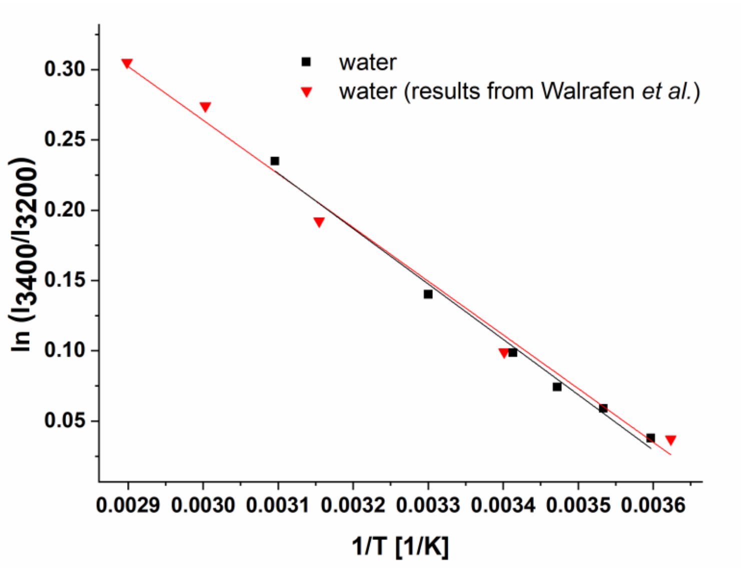

Figure S2: Arrhenius plot of the ratio I_{3400}/I_{3200} determined for water within this study and water data extracted from Walrafen et al. [1].

SI3 Procedure used to estimate the energy barriers for the movement of water molecules in the bulk and close to the Ag surface

The simulations were performed on a 100×100×32 FCC lattice and were averaged over 10^4 time units. The hard wall {100} was placed at $z = 1$ (layer $z = 32$ was also immobile) representing an inert reflecting wall of Ag. In the remaining directions, periodic boundary conditions were used. All lattice sites were occupied either by water-like or by Ag-like elements. A large size in both x and y directions provided good spatial averaging for results. To introduce the electrostatic interactions to the DLL simulation, the probability of movement of the solvent molecule was modified according to the following formula $P = \exp(-E/RT)$, where T is temperature ($T = 300$ K), R is universal gas constant and E is the diffusion/translational activation energy expressed in J/mol. The reference energy level was set to the bulk state (i.e., $E = 0$ for solvent molecules in the bulk or $E = E_{w2} - E_{b2}$ for water molecules close to the Ag surface (immobile wall)). The parameter E_{b2} stands for water translation activation energy (potential barrier) in the bulk and E_{w2} stands for water translation activation energy close to the Ag surface. The index 2 refers to the potential barrier height, while the index 1 stands for (as showed later) the absolute interaction energy (depth of the potential well). It is also worthy to notice that, for the athermal case, all interactions are equal ($P = 1$). Since there are different values in the literature for the interaction energy and, therefore, potential barriers for water translation in the bulk and close to the Ag surface [2-5], four different pairs of E_{w2} and E_{b2} values were considered according to different assumptions.

CASE A

The distance between two water molecules was assumed to be equal to 2.8 Å, as a commonly accepted O···O distance in liquid water [6]. The depth of a single potential

well (Lennard-Jones type potential with the addition of electrostatic interactions [7]) for water was assumed to be equal to E_{b1} = 41 kJ/mol, according to the work of Michaelides [2]. The overlapping of two wells (the green dashed lines on the figures below correspond to constituent potential curves, while the black continuous line represents their sum) with the mentioned parameters leads to the estimated energy barrier in the bulk E_{b2} = 26 kJ/mol as the figure below presents.

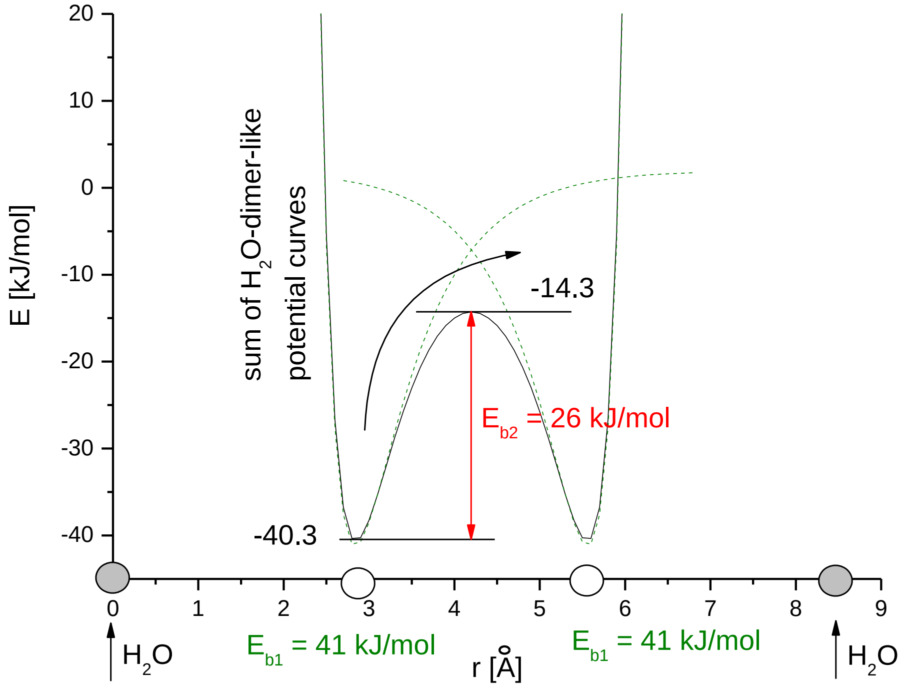

To estimate the E_{w2} value, an analogous procedure was applied assuming that the total energy of the interaction between the water molecule and its nearest neighbours, close to the Ag surface, is E_{w1} = 45 kJ/mol (originally 0.46–0.48 eV) and the distance between the water molecule and the Ag surface is equal to 2.8 Å [2]. These assumptions lead to E_{w2} = 29.3 kJ/mol, as the figure below shows.

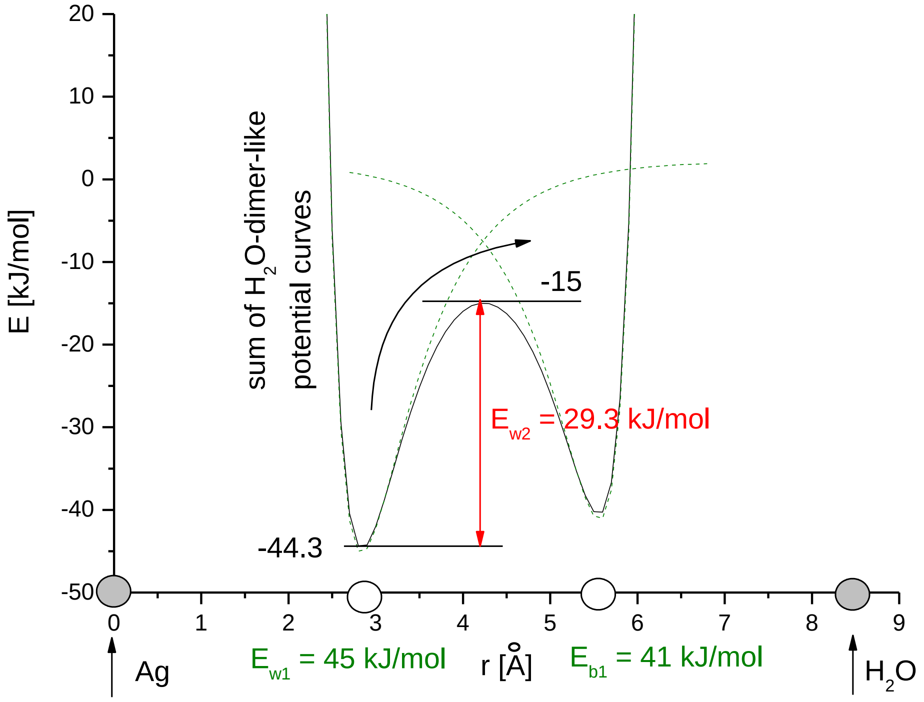

The difference between E_{w2} and E_{b2} is equal to 3.3 kJ/mol and, consequently, the probability of movement of the solvent molecule decreased to $P \approx 0.2663$, comparing with the DLL model with no electrostatic interactions.

CASE B

The second considered approach was also based on the results from Michaelides work, as two approaches to determine the energy values of water–water and water–metal interactions, near the Ag surface, were presented (for details, see Michaelides et al. [2]). Assuming E_{b1} = 36 kJ/mol for bulk water, E_{b2} = 26.6 kJ/mol was found. Consequently, a superposition of two potential wells, with depth values of E_{b1} = 36 kJ/mol and E_{w1} = 45 kJ/mol, leads to E_{w2} = 30.4 kJ/mol, which gives $E = E_{w2} - E_{b2}$ = 7.6 kJ/mol and $P \approx 0.0494$.

CASE C

The next approach is based on the experimental results presented by Holtz et al. [8]. The authors, by using pulsed magnetic field gradient NMR spectroscopy, determined the self-diffusion constant for liquid water at a broad temperature range. Using the Arrhenius-type equation $D = D_0\exp(-E_a/RT)$, the translational activation energy was determined as $E_a = E_{b2}$ = 17.5 kJ/mol. Next, the E_{b1} = 27.5 kJ/mol value was found to be the depth of the potential well for which the energetic barrier E_{b2} reaches an appropriate value. The E_{b1} value was used to estimate E_{w1} according to the formula: $E_{w1} = (3/4E_{b1} + E_{metal\text{–}water})$ = 26.5 kJ/mol, where $E_{metal\text{–}water}$ = 10 kJ/mol as it was found to be the upper value [2] for a Ag–water system by DFT calculations. The factor 3/4 was introduced since water molecules, in the direct vicinity of a metal surface, can be involved in a maximum of three H bonds with other water molecules (one of the H-bond centres of the water molecules is consumed by the water–metal interaction). In the result E_{w2} = 16.6 kJ/mol, $E = E_{w2} - E_{w1} < 0$ and $P = 1$, which corresponds to an athermal case.

CASE D

In the last considered case, E_{b1} = 30 kJ/mol was assumed to be the approximately mean value from the DFT calculations [3] (the potential energy of the well for the biggest water clusters presented in the referred article was taken into account). The calculation procedures presented in detail for the CASE A were applied to estimate E_{b2} = 19 kJ/mol. The values E_{w1}= 32.5 kJ/mol and E_{w2} = 21.1 kJ/mol were obtained according to the procedures presented in the CASE C section. It results in E = 2.1 kJ/mol and $P \approx 0.4309$.

References

1. Walrafen, G. E.; Hokmabadi, M. S.; Yang, W. H. *J. Phys. Chem.* **1988,** *92,* 2433–2438. doi:10.1021/j100320a010
2. Michaelides, A. *Appl. Phys. A: Mater. Sci. Process.* **2006,** *85,* 415–425. doi:10.1007/s00339-006-3695-9
3. Gillan, M. J.; Alfe, D.; Michaelides, A. *J. Chem. Phys.* **2016,** *144,* 130901. doi:10.1063/1.4944633
4. Qin, C. Y.; Whitten, J. L. *J. Phys. Chem. B* **2005,** *109,* 8852–8856. doi:10.1021/jp044067a
5. Ranea, V. A.; Michaelides, A.; Ramírez, R.; Vergés, J. A.; De Andres, P. L.; King, D. A. *Phys. Rev. B: Condens. Matter Mater. Phys.* **2004,** *69,* 205411. doi:10.1103/PhysRevB.69.205411
6. Marechal, Y., Ed. The Hydrogen Bond and the Water Molecule. In *The Water Molecule in (Bio)Macromolecules*; Elsevier, 2007; pp 249–275. doi:10.1016/B978-044451957-3.50011-1
7. Saramak, J.; Halagan, K.; Kozanecki, M.; Polanowski, P. *J. Mol. Model.* **2014,** *20,* 2529. doi:10.1007/s00894-014-2529-5
8. Holz, M.; Heil, S. R.; Sacco, A. *Phys. Chem. Chem. Phys.* **2000,** *2,* 4740–4742. doi:10.1039/b005319h
